# Pulse oximeter saturation target limits for preterm infants: a survey among European neonatal intensive care units

**DOI:** 10.1007/s00431-016-2804-9

**Published:** 2016-11-16

**Authors:** Maurice J. Huizing, Eduardo Villamor-Martínez, Máximo Vento, Eduardo Villamor

**Affiliations:** 1 Department of Pediatrics, Maastricht University Medical Center (MUMC+), P. Debyelaan 25, P.O. Box 5800, 6202 AZ Maastricht, The Netherlands; 2 Division of Neonatology, University & Polytechnic Hospital La Fe, Valencia, Spain

**Keywords:** Oxygen saturation, Preterm, Hypoxia, Hyperoxia

## Abstract

The optimum range of pulse oximeter oxygen saturation (SpO_2_) for preterm infants remains controversial. Between November 2015 and February 2016, we conducted a web-based survey aimed to investigate the current and former practices on SpO_2_ targets in European neonatal intensive care units (NICUs). We obtained valid responses from 193 NICUs, treating 8590 newborns ≤28 weeks per year, across 27 countries. Forty different saturation ranges were reported, ranging from 82–93 to 94–99%. The most frequently utilized SpO_2_ ranges were 90–95% (28%), 88–95% (12%), 90–94% (5%), and 91–95% (5%). A total of 156 NICUs (81%) changed their SpO_2_ limits over the last 10 years. The most frequently reported former limits were 88–92% (18%), 85–95% (9%), 88–93 (7%), and 85–92% (6%). The NICUs that increased their SpO_2_ ranges expected to obtain a reduction in mortality. A 54% of the NICUs found the scientific evidence supporting their SpO_2_ targeting policy strong or very strong.

*Conclusion*: We detected a high degree of heterogeneity in pulse oximeter SpO_2_ target limits across European NICUs. The currently used limits are 3 to 5% higher than the former limits, and the most extreme limits, such as lower below 85% or upper above 96%, have almost been abandoned.

**Table:** 

**What is Known:** • *For preterm infants requiring supplemental oxygen, the optimum range of pulse oximeter oxygen saturation (SpO*_*2*_ *) to minimize organ damage, without causing hypoxic injury, remains controversial*.
**What is New:** • *This survey highlights the lack of consensus regarding SpO*_*2*_ *target limits for preterm infants among European neonatal intensive care units (NICUs). We detected 40 different SpO*_*2*_ *ranges, and even the most frequently reported range (i.e., 90–95%) was used in only 28% of the 193 respondent NICUs*. • *A total of 156 NICUs (81%) changed their SpO*_*2*_ *limits over the last 10 years. The currently used limits are 3 to 5% higher than the former limits, and the most extreme limits, such as lower below 85% or upper above 96%, have almost been abandoned*.

## Introduction

Oxygen is one of the most widely used drugs in the care of (very) preterm infants, and arterial oxygen saturation measured by pulse oximetry (SpO_2_) is the standard, noninvasive, continuous method used to guide oxygen therapy [6, 17, 18]. However, for preterm infants requiring supplemental oxygen, the optimum range of SpO_2_ to minimize organ damage, without causing hypoxic injury, remains controversial [9, 10]. Between 2005 and 2007, five randomized trials, known collectively as the Neonatal Oxygen Prospective Meta-analysis (NeOProM) collaboration, were designed to compare the effects of a lower SpO_2_ target range (85 to 89%) vs. a higher target range (91 to 95%) in preterm infants (<28 weeks) [1, 11]. Combined, these trials suggest that, after postnatal stabilization, the preterm babies assigned to the lower SpO_2_ target range intention to treat may have increased mortality and necrotizing enterocolitis (NEC), while keeping them in the higher SpO_2_ range may increase the risk of retinopathy of prematurity (ROP) [11, 18]. Nevertheless, several neonatologists have raised concerns about potential drawbacks of high SpO_2_ targets and underlined the high degree of conflicting results of the NeOProM trials [2, 4, 7–10, 14]. Additionally, a systematic review and meta-analysis using GRADE criteria concluded that although infants cared for with a higher SpO_2_ target had significantly lower mortality before hospital discharge, the quality of evidence for this estimate of effect is low [5]. Therefore, in the present climate of uncertainty, one would expect institutional variations on SpO_2_ targets.

The purpose of this web-based survey was to investigate how SpO_2_ monitoring policies vary among NICUs in Europe and if the target ranges have been changed in the last years. We also surveyed the expectations of the neonatologists on the effects of changes in SpO_2_ targets and their opinion on the strength of the presently available evidence.

## Methods

This study received approval from the institutional review board of the Maastricht University Medical Center. Potential responders were informed that participation in the survey was voluntary and that completion of the questionnaire implied consent to participate. European regions differ widely in how they organized the care of preterm infants [16], and to the best of our knowledge, an exhaustive list of European NICUs does not exist [3]. Therefore, in order to obtain the maximal number of responses, we contacted through email a delegate of the national neonatology society of each of the countries mentioned below (see “Results”), requesting them to distribute a web-based survey among the NICUs of their respective country. If no response was obtained after 2 weeks, we contacted another neonatologist of the country. The survey was open between October 2015 and February 2016. The head of the NICU, or a delegated senior neonatologist, was required to fill in the survey, which consisted of the following 10 questions:

1. Please indicate the hospital, city, and country where your NICU is placed.
2. What is your professional position? (head of the NICU/senior neonatologist)
3. In the last year, approximately how many infants born at ≤28 weeks gestation have been admitted in your NICU?
4. Does your NICU have a written unit policy or guideline for the desired oxygen saturation range for infants born at ≤28 weeks gestation who are receiving oxygen therapy? (Yes/No)
5. In your NICU, in what range do you try to maintain oxygen saturations for infants born at ≤28 weeks gestation who are receiving oxygen therapy?
6. Has your NICU’s policy regarding oxygen saturation targeting for infants born at ≤28 weeks gestation been changed in the last 10 years? (Yes/No)
7. If your answer to question 6 was “yes”, when were the last changes introduced?
8. If your answer to question 6 was “yes”, indicate the numbers that correspond to the former low and high limits of the oxygen saturation range for a 7-day-old infant born at ≤28 weeks gestation receiving oxygen therapy.
9. If your answer to question 6 was “yes”, which of the following outcomes do you expect to be reduced with the new saturation targeting policy? (Outcomes were mortality, NEC, bronchopulmonary dysplasia, patent ductus arteriosus, ROP, intraventricular hemorrhage, periventricular leukomalacia, and impaired neurodevelopment. Participants were asked to respond through a scale with 1 indicating very improbable and 5 indicating very probable)
10. In your opinion, how strong is the scientific evidence supporting the beneficial/harmful effects of the oxygen saturation targeting policy that you are currently using in your NICU for infants born at ≤28 weeks gestation? (scale with 1 indicating very weak and 5 indicating very strong).

Only descriptive statistics were performed including frequency counts, percentages, mean and standard deviation as well as median and interquartile range (IQR) when appropriate.

## Results

We received 200 responses from European NICUs, of which 193 were valid for analysis. Due to the method of distribution and the absence of an exhaustive list of European NICUs [3, 16], it was not possible to obtain complete data on the number of NICUs that received the survey. Some of the contacted neonatologists distributed the survey in their country but did not notify us of the number of NICUs that were contacted. Therefore, a response rate could not be determined. The 193 respondent NICUs treated 8590 infants aged ≤28 weeks in the last year (median 40, IQR 22–55). The distribution of responses per country was the following: Belgium 9, Bosnia and Herzegovina 1, Bulgaria 1, Czech Republic 3, Denmark 3, Finland 3, France 15, Germany 25, Greece 4, Hungary 6, Iceland 1, Ireland 3, Italy 16, Latvia 1, Norway 1, Portugal 4, Poland 1, Russia 18, Serbia 3, Slovakia 3, Slovenia 1, Spain 27, Sweden 13, Switzerland 6, the Netherlands 9, Turkey 12, and UK 3. No response was obtained from Austria, Cyprus, Estonia, Georgia, Lithuania, Macedonia, Malta, Romania, and Ukraine. The head of the NICU responded the survey in 70% of the cases.

**Fig. 1:**
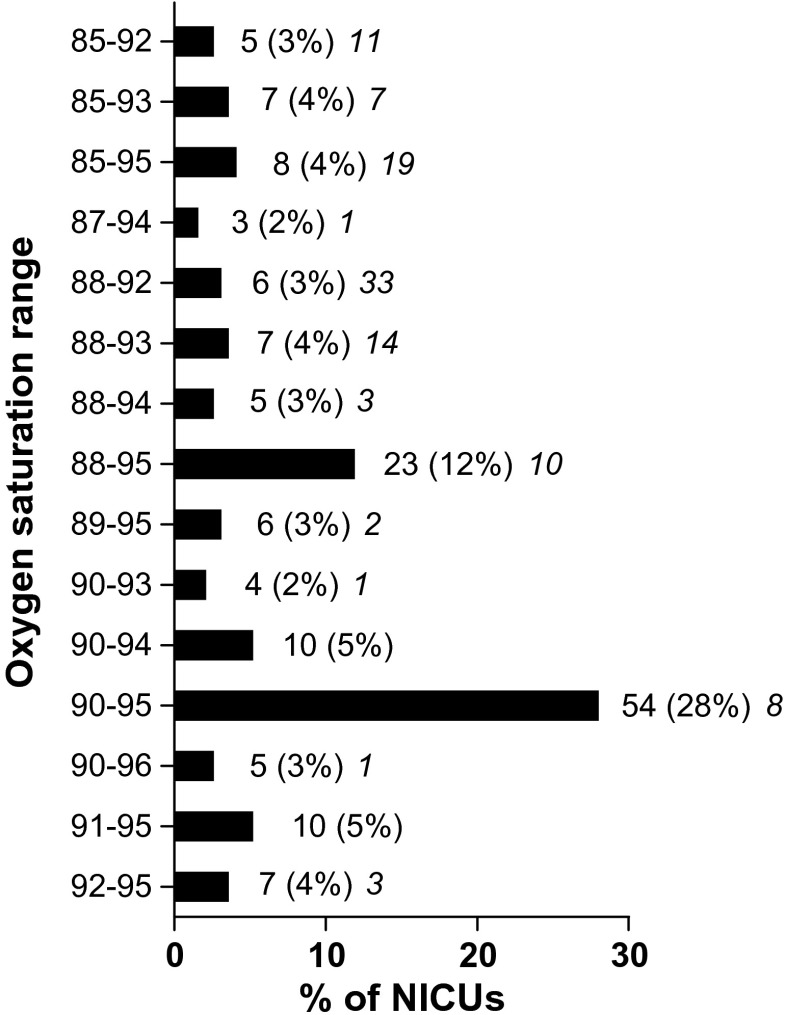
Current SpO_2_ ranges used in 193 European NICUs. Results are expressed as *n* (%). The *number in italic* indicates the number of NICUs having the same range over the last 10 years. For clarity, ranges reported by only one or two NICUs are not shown in the figure. Limits used in only one center are 82–93, 85–97, 86–93, 86–94. 86–95, 86–96, 87–92, 88–97, 89–92, 89–96, 90–92, 90–97, 90–98, 91–96, 92–94, 93–98, and 94–99. Limits used in two centers are 83–93, 85–90, 86–92, 87–93, 87–95, 88–96, 89–94, and 95–96

**Fig. 2:**
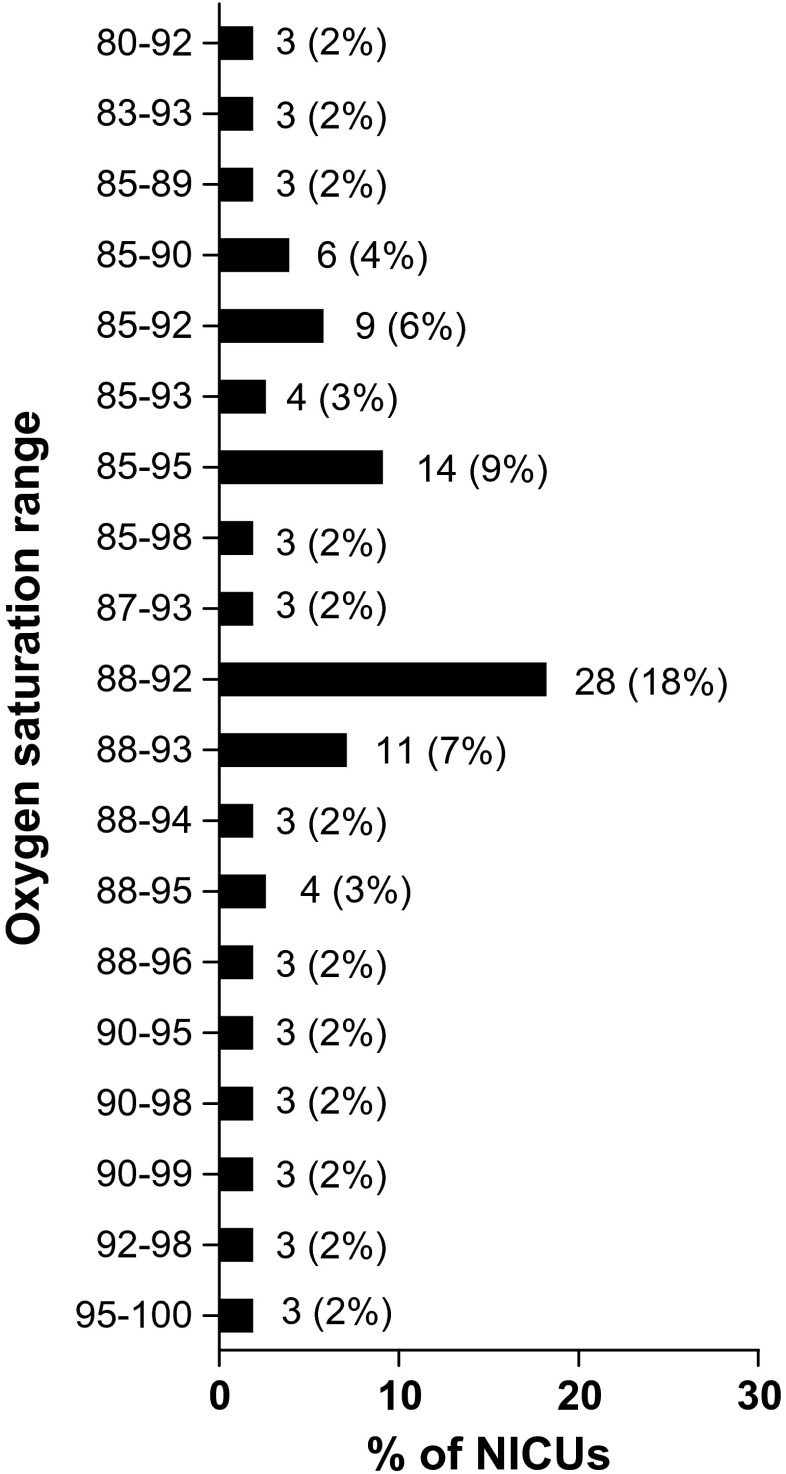
Former SpO_2_ ranges used in 156 European NICUs that changed their limits in the last 10 years. Results are expressed as *n* (%). For clarity, ranges reported by only one or two NICUs are not shown in the figure. Limits used in only one center are 80–87, 84–92, 84–94, 84–96, 85–88, 86–96, 87–95, 88–97, 88–98, 89–95, 89–97, 90–96, 91–100, 92–95, 92–97, 92–99, 93–97, 94–96, 94–98, 94–99, 95–98, 95–99, 92–97, 92–95, 90–100, and 91–100. Limits used in two centers are 80–95, 82–92, 85–94, 86–95, 86–90, 86–93, 86–94, 87–92, and 90–97

**Fig. 3:**
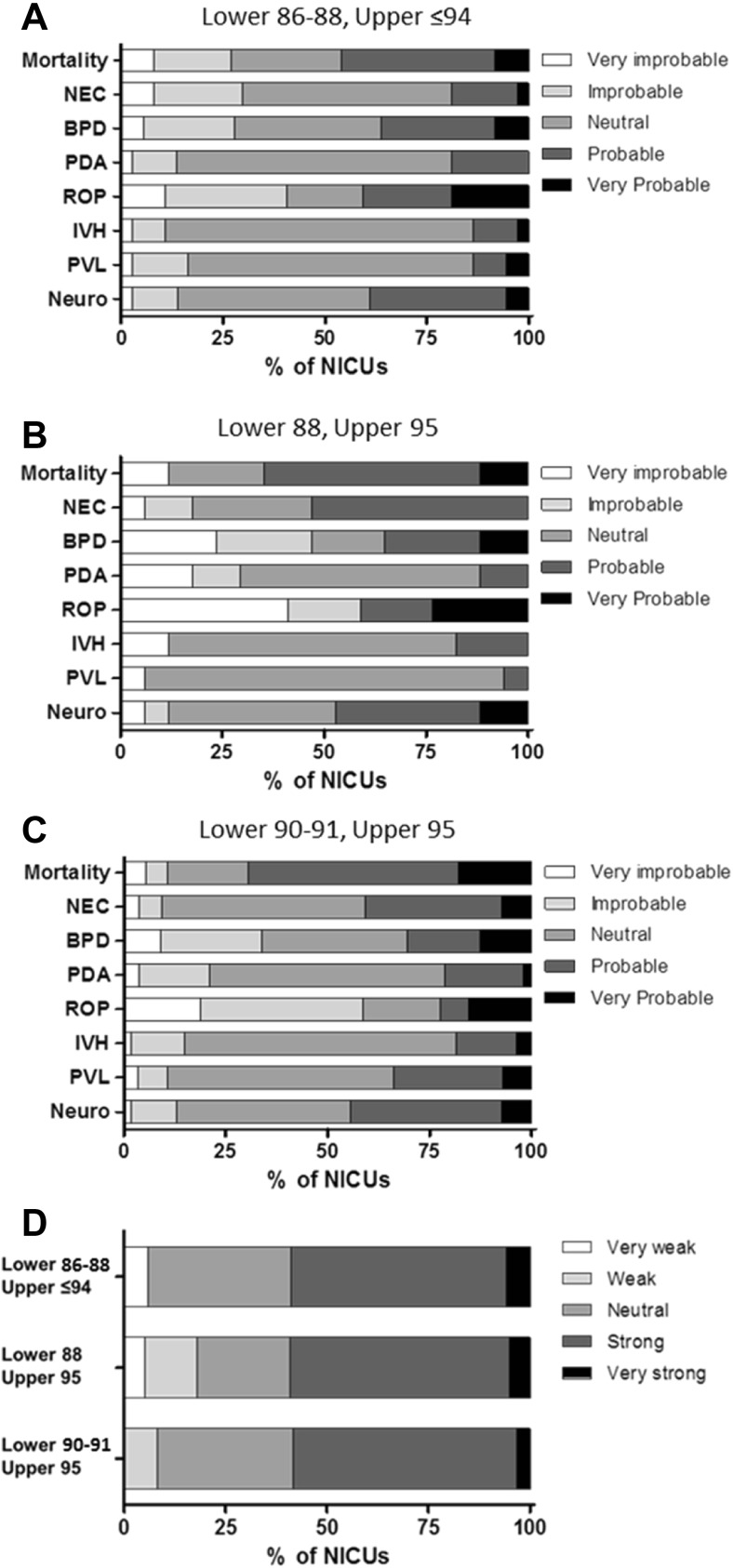
**a**–**c** Expected reduction in adverse outcomes after changes in SpO_2_ ranges. The following question was posed: Which of the following outcomes do you expect to be reduced with the new saturation targeting policy? Participants were asked to respond through a scale with 1 indicating very improbable and 5 indicating very probable. For analysis of this question, responses were divided into three groups: **a** NICUs using a lower limit of 86–88% and an upper limit ≤94% (*n* = 22), **b** NICUs using a range of 88–95% (*n* = 19), and **c** NICUs using a lower limit of 90–91% and an upper limit of 95% (*n* = 55). *Neuro* neurodevelopmental impairment. **d** Opinion on the strength of scientific evidence. The following question was posed: In your opinion, how strong is the scientific evidence supporting the beneficial/harmful effects of the oxygen saturation targeting policy that you are currently using in your NICU for infants born at ≤28 weeks gestation? (scale with 1 indicating very weak and 5 indicating very strong)

A total of 140 NICUs (73%) reported to have a written policy or guideline on oxygen saturation limits. The limits currently used in the 193 NICUs are depicted in Table 1. Limits were combined in 40 different ranges. The four most frequently used ranges were 90–95, 88–95, 90–94, and 91–95% (Fig. 1). The distribution of the ranges across the countries with more than five respondents is shown in Table 2. From the 193 respondent NICUs, 156 (81%) changed their oxygen saturation limits in the last 10 years (9 changed less than 1 year ago, 29 changed 1 year ago, 48 changed 2 years ago, 24 changed 3 years ago, 13 changed 4 years ago, 16 changed 5 years ago, 16 changed more than 5 years ago, and 1 did not answer). The limits formerly used in these 156 NICUs are depicted in Table 1 and the ranges in Fig. 2. Table 3 shows the direction of the changes in SpO_2_ ranges. The expectations on the effect of the changes on outcome are shown in Fig. 3. For analysis of this question, responses were divided into three groups: (1) NICUs using a lower limit of 86–88% and an upper limit ≤94% (*n* = 22), (2) NICUs using a range of 88–95% (*n* = 19), (3) NICUs using a lower limit of 90–91% and an upper limit of 95% (*n* = 55). The evidence supporting the beneficial/harmful effects of their current saturation targeting policy was considered strong or very strong by 54% of the respondents, neutral by 30%, and weak or very weak by 15%. The consideration on the strength of the evidence within the above three groups is depicted in Fig. 3d.

**Table 1:** Current and former SpO_2_ limits used in European NICUs for infants of gestational age ≤28 weeks

	Number of centers	
	Current (*n* = 193), *n* (%)	Former (*n* = 156), *n* (%)
Lower limit		
80		4 (2)
82	1 (1)	2 (1)
83	1 (1)	4 (2)
84		3 (2)
85	12 (8)	53 (27)
86	5 (3)	10 (5)
87	5 (3)	9 (5)
88	33 (21)	65 (34)
89	9 (6)	3 (2)
90	70 (45)	20 (10)
91	10 (6)	2 (1)
92	7 (5)	8 (4)
93		2 (1)
94	1 (1)	3 (2)
95		5 (3)
Upper limit		
87		1 (1)
88		1 (1)
89		3 (2)
90	1 (1)	9 (5)
92	8 (5)	53 (27)
93	18 (12)	31 (16)
94	21 (14)	9 (5)
95	92 (60)	47 (24)
96	11 (7)	8 (4)
97	2 (1)	6 (3)
98		14 (7)
99	1 (1)	6 (3)
100		5 (3)

The former limits refer to the limits previously utilized by the 156 NICUs that changed their limits in the last 10 years

**Table 2:** Distribution of SpO_2_ targeting limit ranges per country

Country (respondents)	Centers that changed, *n* (%)	Number of ranges	Most frequent ranges (%)		
			First	Second	Third
Spain (*n* = 27)	24 (89)	12	90–95 (44)	88–95 (15)	90–94 (7)
Germany (*n* = 25)	16 (64)	12	90–95 (20)	85–95 (16)	85–93 (12)
Russia (*n* = 18)	17 (94)	13	90–95 (28)	92–95 (11)	
Italy (*n* = 16)	9 (56)	12	88–95 (19)	90–95 (13)	87–94 (13)
France (*n* = 15)	12 (80)	9	88–95 (33)	92–95 (13)	89–95 (13)
Sweden (*n* = 13)	10 (77)	6	90–94 (23)	90–95 (23)	91–95 (23)
Turkey (*n* = 12)	9 (75)	5	90–95 (67)		
Netherlands (*n* = 9)	9 (100)	7	90–95 (22)	91–95 (22)	
Belgium (*n* = 9)	7 (78)	8	88–95 (22)		
Hungary (*n* = 6)	6 (100)	3	90–95 (67)		
Switzerland (*n* = 6)	4 (67)	5	85–92 (33)

**Table 3:** Changes in SpO_2_ targeting limits introduced in European NICUs in the last 10 years

Lower limit	Upper limit		
	Increased	Maintained	Decreased
Increased	*n* = 77 (40%)	*n* = 28 (15%)	*n* = 13 (7%)
	L: 90 (85–92)	L: 90 (85–92)	L: 88 (86–94)
	U: 95 (92–97)	U: 95 (92–96)	U: 95 (92–99)
Maintained	*n* = 3 (2%)	*n* = 37 (23%)	*n* = 4 (2%)
	L: 88 (85–88)	L: 88 (83–93)	L: 90 (88–90)
	U: 95 (90–96)	U: 95 (90–98)	U: 95 (92–96)
Decreased	0	*n* = 1 (1%)	*n* = 30 (16%)
		L: 85	L: 88 (82–92)
		U: 93	U: 94.5 (92–97)

*n* = number of centers (%). The number after L and U represents the median (range) of the current SpO_2_ targeting limit. For example, 77 centers have increased both the lower and the upper SpO_2_ targeting limit in the last 10 years. These 77 centers currently used a median lower limit of 90% (range 85–92) and a median upper limit of 95% (range 92–97)

*L* lower limit, *U* upper limit

## Discussion

This survey highlights the lack of consensus regarding SpO_2_ target limits for preterm infants among European neonatologists. We detected 40 different SpO_2_ ranges and even the most frequently reported range (i.e., 90–95%) was used in only 28% of the respondent NICUs. The heterogeneity in SpO_2_ target ranges was also a general observation within countries, since only in two cases (Turkey and Hungary) the same range was used by more than 50% of the NICUs of the same country. A similar degree of variability in SpO_2_ targets has been recently reported among the NICUs of the UK [12]. Although the method used for the distribution of our survey does not allow us to calculate a response rate, we obtained numerous responses of the majority of European countries, including the largest NICUs. Other surveys on European NICUs reported a number of responses similar to the one obtained by us [3]. Therefore, we believe the present results are representative of current management practices in Europe.

An 81% of the NICUs reported a recent change in their policy of SpO_2_ target limit. Despite the variability, two consequences of these changes can be identified. First, the currently used limits are 3 to 5% higher than the former limits. Consequently, it would be wise to scrutinize the incidence in conditions associated with hyperoxia such as ROP in the coming years. Second, the most extreme limits, such as lower below 85% or upper above 96%, have almost been abandoned. Taken together, these changes may reflect the impact of the evidence generated by the NeOProM trials on clinical practice. It has been argued that, at present, the most rigorously evaluated evidence is that targeting a SpO_2_ of 91 to 95% is safer than targeting a SpO_2_ of 85 to 89% [11, 15]. In 2013, a European panel of experts, convened under the auspices of the European Association of Perinatal Medicine to update evidence-based guidelines on the management of neonatal respiratory distress syndrome, recommended a saturation range of 90–95% [13]. Our survey shows that this recommendation is followed by only 28% of the respondent NICUs. Moreover, the NeOProM higher target of 91–95% has been adopted by only 5% of the NICUs. In general, the NICUs that changed their range to 90–95 or 91–95% expect to obtain a reduction in mortality and, to a lesser extent, a reduction in neurodevelopmental impairment and NEC.

A number of neonatologists consider that the NeOProM upper limits may result in partial pressures of oxygen above the physiologic limits given the characteristics of the hemoglobin oxygen saturation curve [9, 10]. They suggest that it is safer to widen the target, using SpO_2_ ranges like 86–93, 87–94, or 88–94% [9, 10]. Our survey shows that these intermediate targets are currently used by 14% of the respondent NICUs. It is noteworthy that 45% of the NICUs that changed to intermediate saturation targets also consider achieving a reduction in mortality probable or very probable. Finally, a 12% of the respondent NICUs are using a wider SpO_2_ range of 88–95%. Their expectations are similar than the ones reported by the NICUs using the higher limits. Interestingly, independently of their choice of saturation targets, more than 50% of the respondents consider the evidence supporting their choice as strong or very strong.

In conclusion, a definitive answer to the optimal SpO_2_ level for preterm neonates is still elusive. This is reflected in a wide variation of unit policies on SpO_2_ targets across Europe and highlights the necessity of further investigation. Moreover, since all five NeOProM trials used a similar study design, a prospective meta-analysis is planned when follow-up of study infants has occurred in the last trial [1]. Hopefully this meta-analysis will help to find the best evidence-based SpO_2_ targets.

## Abbreviations

IQR: Interquartile range
NEC: Necrotizing enterocolitis
NeOProM: Neonatal Oxygen Prospective Meta-analysis
NICU: Neonatal intensive care unit
ROP: Retinopathy of prematurity
SpO_2_: Oxygen saturation
